# Prediction improvement of potential PV production pattern, imagery satellite-based

**DOI:** 10.1038/s41598-020-76957-8

**Published:** 2020-11-17

**Authors:** A. Ben Othman, K. Belkilani, M. Besbes

**Affiliations:** 1 Laboratory of Robotics, Informatics and Complex Systems, National School of Engineers of Tunis, University Tunis El Manar, Tunis, Tunisia; 2 Higher Institute of Information and Communication Technologies, University of Carthage, Tunis, Tunisia; 3 National School of Engineers of Carthage, University of Carthage, Tunis, Tunisia

**Keywords:** Energy science and technology, Mathematics and computing

## Abstract

The results obtained by using an existing model to estimate global solar radiation (GHI) in three different locations in Tunisia. These data are compared with GHI meteorological measurements and PV_Gis satellite imagery estimation. Some statistical indicators (R, R^2^, MPE, AMPE, MBE, AMBE and RMSE) have been used to measure the performance of the used model. Correlation coefficient for the different stations was close to 1.0. The meteorology and satellite determination coefficient (R^2^) were also near 1.0 except in the case of Nabeul station in which the meteorology measurements (R) were equals to 0.5848 because of the loss of data in this location due to meteorological conditions. This numerical model provides the best performance according to statistical results in different locations; therefore, this model can be used to estimate global solar radiation in Tunisia. The R square values are used as a statistical indicator to demonstrate that the model’s results are compatible with those of meteorology with a percentage of error less than 10%.

## Introduction

Knowledge of local solar radiation is essential for many applications. Despite the importance of solar radiation measurements, this information’s source is not available due to the high cost of the sensors and it needs of continuous maintenance and calibration requirements^1–3^. The limited coverage of radiation values dictates the need to develop models to estimate solar radiation based on other more readily available, data^4–8^. The aim of the appraisal is to specify and classify sum of sites in Tunisia^9–12^. This evaluation is important to have significant data on which the development model can be based to furnish a broad roadmap for coming project developed^13–15^. Generally, the solar data on-ground needs the use of meteorological stations for 10 years.

The main factors were considered in this approach such as water resources, economic, costs, and environmental considerations^16–18^.

Experiences and Investigations takes away for the last thirteen years have shown that it require at length eleven years of solar data to predict the values of the global solar radiation^19–22^. This signifies that it is infeasible to construct PV plants in few years because weather stations are not capable of provides data that cover 11 years^23–25^. Furthermore, that satellite imagery of radiation is not sufficient for the selection. A lot of PV plant installation methods were developed to solar assessment that help to create a numerical model^26–29^. This method provides the values for different locations using defined parameters like latitude, longitude, and other specific parameters^30–32^.

The objective of this study was to validate a model used in prediction monthly global solar radiation on a horizontal surface. This validation based on a comparison between measured data provided by the National Institute of Meteorology of Tunisia (NIM_ Tunisia) and the GHI estimates from satellite PV_Gis. several stations were selected: Bizerte in Northern of Tunisia, Nabeul in the northern east of Tunisia and Djerba in the southern of Tunisia. The results show that the established model can be favorably used to calculate the GHI for all the seasons of year and all days in any location.

The commonly used model yields good results using the main meteorological and physical parameters: the extraterrestrial radiation, the hour angle, the atmospheric optical distance, the elevation, and the latitude. In our model, a good congruence was verified at the three stations with an error percentage less than 10%. Moreover, while most estimation models of monthly global solar radiation use the sunshine ratio, the persistent challenge for them is that well-functioning methods to determine clear sky global irradiance are still unavailable. Therefore, we propose this model as an efficient approach to predict the global solar radiation all over Tunisia. Its efficiency rests in its capacity to provide accurate measurements at all stations because it can be calibrated to estimate the data in all Tunisian locations and yield good results close to those generated by meteorological stations and satellite imagery^33–35^.

The strength of this study lies in being the first in Tunisia to provide an approach of generating local solar radiation using a numerical model. Thus, its results make a great contribution to the literature, the applications that require solar radiation data and the applications used in solar energy production.

## Materials and sources

### Instrument of measurement of NIM of Tunisia

The pyranometer is a radiometer designed for measuring the GHI, provides experimental data. The received flux is converted to heat by the blackened surface. The variance of the temperature between the surface and the instrument is in proportion to the irradiance of the GHI. It can be detected by a “Thermopile” that consists of a number of thermocouple junctions, usually joined together in series^36,37^.

In this section of study, we use data for several stations Bizerte, Nabeul and Djerba. In this location kipp_Zonen pyranometer is used for different measurements.

### Solar radiation estimates from PV_Gis satellite imagery

**Figure 1:**
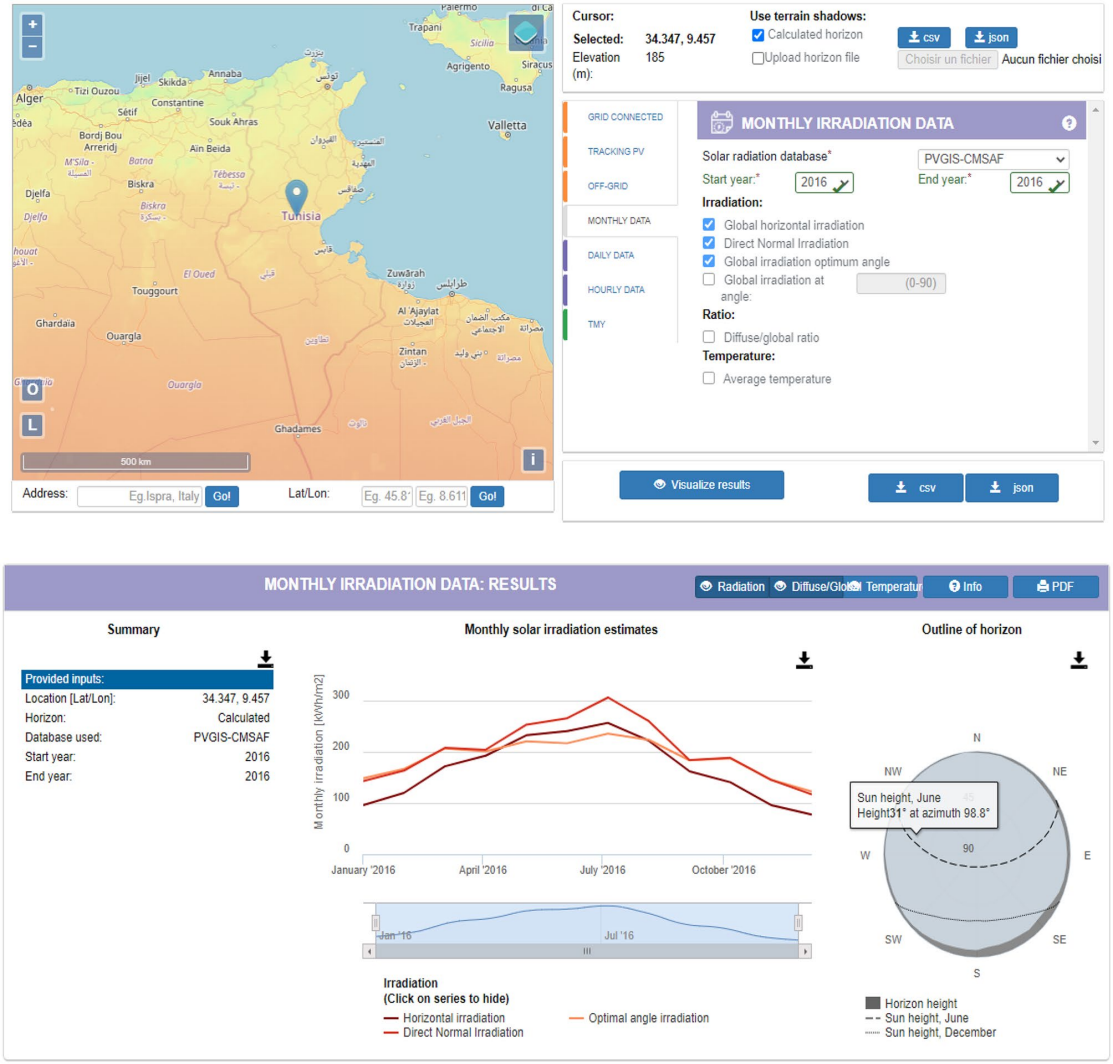
PVGIS satellite imagery’s interface. Source: https://ec.europa.eu/jrc/en/PVGIS/docs/methods.

We present in this section the results of the prediction of the solar radiation from satellite imagery PV_GIS^38^. PVGIS provide an accurate solar radiation free database for Europe, Africa Mediterranean, and South-West Asia. It provides yearly average irradiation as well as the sum of the average sum of GHI per square meter received by the modules of the system in KWh/m^2^. Moreover, it must be highlighted that the uncertain of measurement in the spatial interpolation of ground station data in several locations is present. This is caused by the distance between different station and the local climatic conditions (Fig. 1).

## Numerical simulation

To validate the result and to achieve closeness between the DNI provided by satellite imagery and meteorology, a numerical model is detailed to predict the global solar radiation.

### Global solar radiation estimation

The basic solar radiation equations and the empirical relations are used in this section to give the GHI as a result. The very important equations are detailed in^29,39^. They determine the solar irradiance $G_{i,\gamma }$ incident on a PV or solar panel array inclined with an angle $i$ and oriented with an angle $\gamma$ for the south direction. Using the declination $\delta$ and the hour angle $w$ we can calculate the sun position:

$$\delta =23.45\times \sin\left[360{\frac{\left(284+n\right)}{365}}\right]$$

1 $$\omega ={t}_{s}\times \frac{360}{24}$$

where $n$ is an integer representing the number of the day $n\in \left[\mathrm{0,365}\right]$ , $t_{s}$ is the Solar time:

2 $$\begin{aligned} {t}_{s}&={t}_{l}+N\pm {t}_{seas}\pm \frac{1}{15}L\\&+\frac{1}{60}\left[9.9\mathrm{sin}\left[A-7.7\mathrm{sin}(B)\right]\right]-12 \end{aligned}$$

where A = 2(0.986n + 100) and B = 0.986n−2, where $t_{l}$ is the legal time, *N* is the time zone,$t_{seas}$ is the seasonal correction, and *L* being the longitude.

The sun elevation angle $\alpha_{a}$, its azimuth angle $a$ and the sunlight duration $D_{j}$ satisfy the Eq. (3)

$$\mathrm{sin}{\alpha }_{a}=\mathrm{sin}\delta \times \mathrm{sin}\varphi +\mathrm{cos}\delta \times \mathrm{cos}\varphi \times \mathrm{cos}\omega$$

$$\mathrm{sin}a=\frac{\mathrm{sin}\omega \mathrm{cos}\delta }{\mathrm{cos}{\alpha }_{a}}$$

3 $${D}_{j}=\frac{2}{15}{\mathrm{cos}}^{-1}\left(-\mathrm{tan}\delta \mathrm{tan}\varphi \right)$$

where $\varphi$ present the latitude. The solar global irradiance ${G}_{i,\gamma }$ incident on a photovoltaic array is composed of the direct irradiance ${S}_{i,\gamma }^{*}$ and of the diffuse ${D}_{i}^{*}$ :

4 $${G}_{i,\gamma }={S}_{i,\gamma }^{*}+{D}_{i}^{*}$$

The solar direct irradiance ${S}_{i,\gamma }^{*}$ is expressed by

5 $${S}_{i,\gamma }^{*}={I}_{0}^{*}\left[\mathrm{cos}\left({\alpha }_{a}\right)\times \mathrm{sin}\left(i\right)\mathrm{cos}\left(a-\gamma \right)+\mathrm{sin}\left({\alpha }_{a}\right)\times \mathrm{cos}\left(i\right)\right]$$

where ${I}_{0}^{*}$ is the Direct irradiance,

6 $${I}_{0}^{*}=1353 \times exp\left[-\frac{m{T}_{L}}{0.9m+9.4}\right]$$

with $m$ being the atmospheric optical distance.

$m=\frac{C}{sin{\alpha }_{a}}$ where c = 1–0.1z and ${\alpha }_{a}>15^\circ$ at the $z$ altitude and ${T}_{L}$ is the Linke turbidity factor. The diffuse irradiance ${D}_{i}^{*}$ can be given by:

7 $${D}_{i}^{*}=\frac{1+\mathrm{cos}i}{2}{D}_{0}^{*}+\frac{1-\mathrm{cos}i}{2}{a}_{1}{G}_{0}^{*}$$

with ${a}_{1}$ being the albedo, ${D}_{0}^{*}$ being the atmospherical diffuse irradiance on a horizontal surface, and ${G}_{0}^{*}$ being the global solar irradiance:

$${D}_{0}^{*}=\frac{1353}{25}{\left(\mathrm{sin}{\propto }_{a}\right)}^{1/2}\left[{T}_{L}-0.5-{\left(\mathrm{sin}{\alpha }_{a}\right)}^{1/2}\right]$$

8 $${G}_{0}^{*}=\left(1270-56{T}_{L}\right){\left(\mathrm{sin}{\alpha }_{a}\right)}^{(T_L+36)/33}$$

## Results and discussion

Experimentations were carried out in three different positions in Tunisia.

Different results are validated with those given by the meteorological station and the satellite imagery.

A good congruence between the experiment, meteorology, and satellite imagery for the GHI is found (See Tables 1, 2 and 3).

**Table 1:** GHI values of Bizerte station.

Months	GHI (Kwh/m^2^)			
	Numerical model	Satellite	Meteorology	Number of measured days
Jan	69	72	68	31
Feb	76	88	74	28
Mar	126	149	119	29
Apr	182	173	179	28
May	220	212	215	31
Jun	248	235	240	30
Jul	239	247	239	30
Aug	214	216	208	31
Sep	126	160	137	27
Oct	98	125	88	26
Nov	79	79	77	30
Dec	69	65	71	31

**Table 2:** GHI values of Nabeul station.

Months	Global solar radiation (Kwh/m^2^)			
	Numerical model	Satellite	Meteorology	Number of measured days
Jan	80	82	79	28
Feb	96	98	82	27
Mar	158	161	130	29
Apr	182	182	193	30
May	220	223	211	30
Jun	228	239	193	30
Jul	231	250	121	16
Aug	221	221	39	6
Sep	160	161	155	29
Oct	130	132	101	28
Nov	89	90	82	28
Dec	75	75	31	13

**Table 3:** GHI values of Djerba station.

Months	Global solar radiation (Kwh/m^2^)			
	Numerical model	Satellite	Meteorology	Number of measured days
Jan	103	103	108	31
Feb	120	122	115	28
Mar	170	183	172	31
Apr	201	202	203	30
May	229	232	234	31
Jun	230	243	233	30
Jul	229	254	229	30
Aug	203	229	203	30
Sep	170	177	171	30
Oct	134	150	134	31
Nov	97	108	97	30
Dec	89	93	89	31

**Figure 2:**
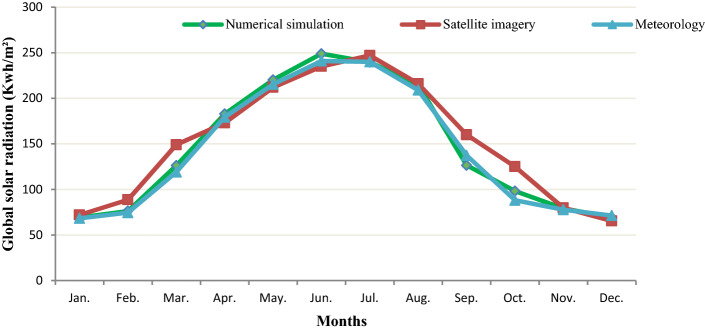
GHl for Bizerte using meteorology data measured, satellite estimation and numerical model’s result.

**Figure 3:**
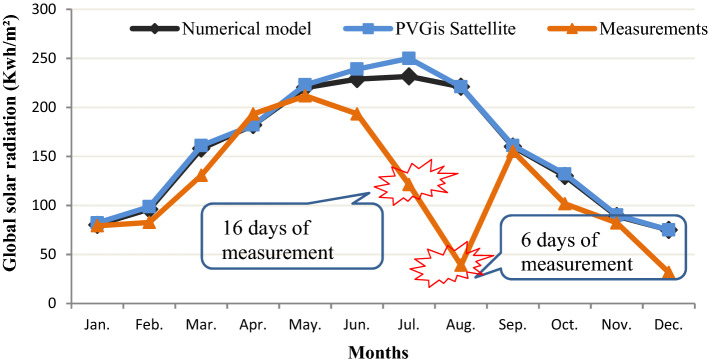
GHI for Nabeul station using Meteorology data measured, satellite estimation and numerical model’s result.

The congruence is verified also in Figs. 2 and 3, which show the calculated values of monthly measured GHI, meteorological and satellite of the different locations. They show the good results obtained by the GHI numerical model. It can be deducted that the maximum monthly average daily GHI 248.92 KWh/m^2^ was in Bizerte station in June. This explains the highest amount of bright sunshine attainment in summer.

Figure 3 represent the GHI for Nabeul station from January, 1 to December, 31 It shows that the numerical model gives a good estimation for the whole year only for July in which the measurement from meteorology station of Nabeul are taken during 16 days and August in which the measurements were taken over 6 days. This explains the role of numerical simulation to correct the messing data and to validate the unavailable data for any location in Tunisia from the year.

## The evaluation of the performance

**Figure 4:**
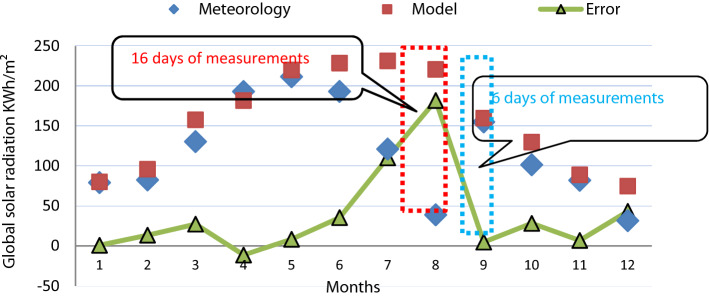
Difference between estimated and measured GHI. Data registered at the station of Nabeul.

According to Fig. 4 we have in the same diagram curves that represent the measured values given by the NIM of Tunisia and the results estimated by the numerical model and the mean errors between the measured data and those calculated by the model. Error values range between 2 and 10%.

Principally, this incongruity is mostly due, first to the lack of data in case of impossibility for the meteorological pyranometer’s failure to provide the right measurement such as in Nabeul station. In July the measurement was received all over 16 days and in August they were taken in 6 days. Second, this is justified by the inaccuracy and errors in the mentioned data of radiation and the temperature degrees from the location.

The performance of the model I evaluated by comparing the calculated global solar radiation with the measured data and those estimated by the satellite imagery.

Several statistical tests were used to control the validation and the goodness of the regression models in terms of the coefficient of determination.

To check the relation between measured and estimated data, we generally use a statistical method by calculating the coefficient of determination $\left({R}^{2}\right)$, which can be expressed by the following equation:

9 $${R}^{2}=\frac{{\sum }_{i=1}^{n}{\left({G}_{i,m}-{G}_{i,c}\right)}^{2}}{{\sum }_{i=1}^{n}{\left({G}_{i,m}-\stackrel{-}{{G}_{i,c}}\right)}^{2}}=1-\frac{{RMSE}^{2}}{{\sigma }^{2}}$$

where $\left({G}_{i,m}\right)$,$\left({G}_{i,c}\right)$,$\left(\stackrel{-}{{G}_{i,m}}\right)$, RMSE are the measured GHI ,the calculated GHI, the average of the measured GHI, and the root mean square error^40,41^.

The root mean square of $\left({R}^{2}\right)$, is the correlation coefficient (R) which is a linear correlation coefficient that returns a value of between − 1 and + 1 we can so deduce if a good linear relationship between measured and estimated data exist or not (case of R = 0).

To prove the agreement between the measured and calculated values, we can evaluate the percent error (PE) which is mentioned below. Thus the results of the PE calculations remain between threshold values that indicate the validity of the models. The (MPE) is obtained by the sum of the PE values and the number of observations. The absolute of the MPE value is designated as Mean Absolute Percent Error (MAPE).

10 $$PE=\left(\frac{{G}_{i,m}-{G}_{i,c}}{{G}_{i,m}}\right)\times 100$$

11 $$MPE=\frac{1}{n}\sum_{i=1}^{n}\left(\frac{{G}_{i,m}-{G}_{i,c}}{{G}_{i,m}}\right)\times 100$$

12 $$MAPE=\frac{1}{n}\sum_{i=1}^{n}\left(\left|\frac{{G}_{i,m}-{G}_{i,c}}{{G}_{i,m}}\right|\right)\times 100$$

In addition to these later equations, statistical errors are generally used to calculate the regression model, which are Mean Biased Error (MBE), Mean Absolute Biased Error (MABE) and Root Mean Square Error (RMSE) (see Table 4).

**Table 4:** Statistical results of the monthly GHI for different stations.

Station		PE	MAPE	MPE	MBE	MABE	RMSE	R^2^	R
Bizerte	Satellite	− 6,3434	5,6604	6,3434	5,9504	11,9339	15,6853	0,9562	0,9779
	Meteorology	1,6337	10,2852	− 1,6337	− 2,5278	4,7520	5,9096	0,9940	0,9970
Nabeul	Satellite	− 1,9921	1,9921	1,9921	3,5667	3,5667	6,2826	0,9948	0,9974
	Meteorology	22,1478	23,1826	− 22,1478	− 37,4917	39,3750	64,8224	0,3419	0,5848
Djerba	Satellite	0,5848	5,9684	5,9684	9,9667	9,9667	13,0264	0,9813	0,9906
	Meteorology	− 0,5197	1,3158	0,5197	0,9667	2,0000	2,7638	0,9978	0,9989

13 $$MBE=\frac{1}{n}\sum_{i=1}^{n}\left({G}_{i,m}-{G}_{i,c}\right)$$

14 $$MABE=\frac{1}{n}\sum_{i=1}^{n}\left(\left|{ G}_{i,m}-{G}_{i,c}\right|\right)$$

15 $$RMSE=\frac{1}{n}\sqrt{\sum_{i=1}^{n}{\left({G}_{i,m}-{G}_{i,c}\right)}^{2}}$$

### Comparison between the measured and the estimated values of global solar radiation

#### Bizerte station

The correlation coefficient of the evaluated meteorology and satellite imagery are closer to 1.0. The highest correlation coefficient of the global solar radiation is obtained as 0.9970 with meteorology data, while for satellite imagery the value is obtained as 0.9779. It indicates that our model has good agreement with meteorology rather than satellite imagery. The lowest values of the statistical analysis are obtained as PE (1.6337) MPE (− 1.6337) MBE (− 2.5278) MABE (4.7520) RMSE (5.9096).

#### Nabeul station

The correlation coefficient of the meteorology and the satellite imagery data at Nabeul station is acquired as 0.5848 (the lowest) and 0.9974 (the highest) respectively. The lowest MAPE (1.9921), MPE (1.992), MBE (3.5667), MABE (3.5667), RMSE (6.2628) are obtained from satellite imagery while meteorology gives the highest value due to the loss of the measurement.

#### Djerba station

The lowest and highest values of correlation coefficient between calculated and measured global solar radiation at Djerba station are obtained from satellite imagery (0.9906) and for meteorology (0.9989). The optimal PE is obtained from meteorology (− 0.5197). The required ideal values of the MAPE, MPE, MBE, RMSE from meteorology such as 1.3158, 0.5197, 0.9667, 2.000, 2.7638, respectively.

Results from three chosen stations indicate that the model better agrees with meteorology data than satellite imagery. They are indicative for the performance of the model for estimating GHI in different locations in Tunisia.

**Figure 5:**
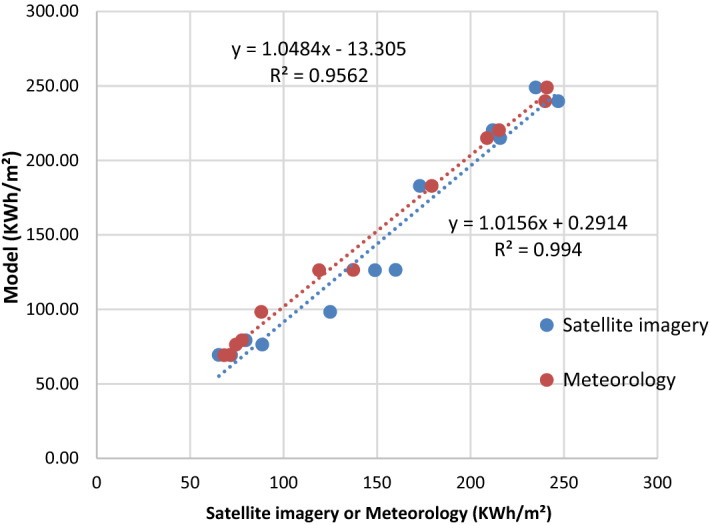
Comparison between measured and estimated GHI at the station of Bizerte.

**Figure 6:**
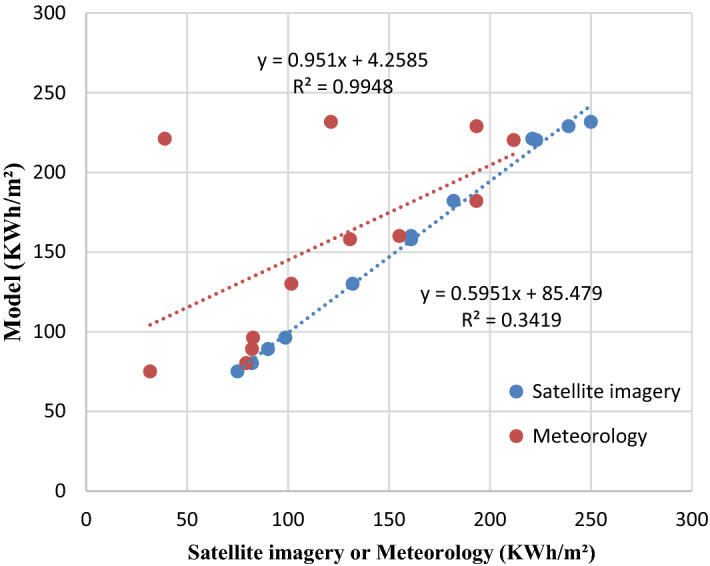
Comparison between measured and estimated GHI at the station of Nabeul.

**Figure 7:**
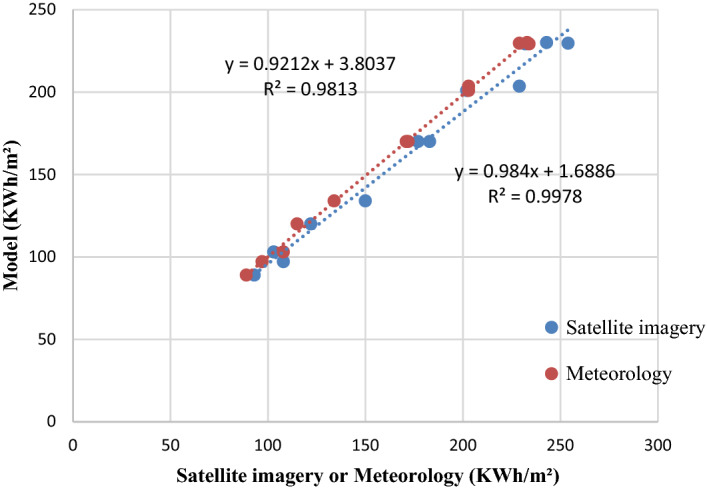
Comparison between measured and estimated GHI at the station of Djerba.

The goodness of the estimation of GHI shown in Figs. 5, 6 and 7. These statistical results can explain the performance of the used model for estimation.

## Conclusion

The global solar radiation measured by pyranometers must be corrected for the obscured part of the sky and for the technical problem due to the use of electronic sensors. The obtained results through simulation of numerical model give better estimation of global solar radiation.

We can deduce that with these conditions, errors for predicted GHI did not surpass 10%. For the used model is highly accurate for estimating global solar radiation at all Tunisian sites to help researcher to choose the right location to install more PV system efficient.
